# When first line treatment of neonatal infection is not enough: blood culture and resistance patterns in neonates requiring second line antibiotic therapy in Bangui, Central African Republic

**DOI:** 10.1186/s12887-021-02911-w

**Published:** 2021-12-13

**Authors:** Andrea Nebbioso, Oluwakemi F. Ogundipe, Ernestina Carla Repetto, Calorine Mekiedje, Hugues Sanke-Waigana, Gilles Ngaya, Brecht Ingelbeen, Julita Gil-Cuesta

**Affiliations:** 1 Médecins Sans Frontières - Operational Centre Brussels (MSF-OCB), Rue de l’Arbre Bénit 46, 1050 Brussels, Belgium; 2 Institut Pasteur Bangui, CAR, Bangui, Central African Republic; 3 Department of Public Health, Institute of Tropical Medicine, Antwerp, Belgium

**Keywords:** Antibiotic resistance, Neonatal sepsis, Neonatal infection, Neonatal intensive care unit, Blood culture, *Klebsiella*, *Escherichia coli*, Gram-negative bacteria, Central-African Republic, Sub-Saharan Africa.

## Abstract

**Background:**

Infectious diseases account for the third most common cause of neonatal deaths. Globally, antibiotic resistance (ABR) has been increasingly challenging neonatal sepsis treatment, with 26 to 84% of gram-negative bacteria resistant to third-generation cephalosporins. In sub-Saharan Africa, limited evidence is available regarding the neonatal microbiology and ABR. To our knowledge, no studies have assessed neonatal bacterial infections and ABR in Central-African Republic (CAR). Therefore, this study aimed to describe the pathogens isolated and their specific ABR among patients with suspected antibiotic-resistant neonatal infection admitted in a CAR neonatal unit.

**Methods:**

This retrospective cohort study included neonates admitted in the neonatal unit in Bangui, CAR, from December 2018 to March 2020, with suspected antibiotic-resistant neonatal infection and subsequent blood culture. We described the frequency of pathogens isolated from blood cultures, their ABR prevalence, and factors associated with fatal outcome.

**Results:**

Blood cultures were positive in 33 (26.6%) of 124 patients tested (17.9% for early-onset and 46.3% for late-onset infection; *p* = 0.002). Gram-negative bacteria were isolated in 87.9% of positive samples; with most frequently isolated bacteria being *Klebsiella pneumoniae* (39.4%), *Escherichia coli* (21.2%) and *Klebsiella oxytoca* (18.2%). All tested bacteria were resistant to ampicillin. Resistance to third-generation cephalosporins was observed in 100% of tested *Klebsiella pneumoniae*, 83.3% of isolated *Klebsiella oxytoca* and 50.0% of tested *Escherichia coli*. None of the tested bacteria were resistant to carbapenems. Approximately 85.7 and 77.8% of gram-negative tested bacteria were resistant to first-line (ampicillin-gentamicin) and second-line (third-generation cephalosporins) treatments, respectively. In hospital mortality, adjusted for blood culture result, presence of asphyxia, birth weight and sex was higher among neonates with positive blood culture (adjusted relative risk [aRR] = 2.32; 95% confidence interval [CI] = 1.17–4.60), male sex (aRR = 2.07; 95% CI = 1.01–4.26), asphyxia (aRR = 2.42; 95% CI = 1.07–5.47) and very low birth weight (1000–1499 g) (aRR = 2.74; 95% CI = 1.3–5.79).

**Conclusion:**

Overall, 77.8% of confirmed gram-negative neonatal infections could no longer effectively be treated without broad-spectrum antibiotics that are not routinely used in sub-Saharan Africa referral hospitals. Carbapenems should be considered an option in hospitals with surveillance and antibiotic stewardship.

**Supplementary Information:**

The online version contains supplementary material available at 10.1186/s12887-021-02911-w.

## Background

Neonatal sepsis and other neonatal infections have a high disease burden globally [1]. Neonatal mortality is globally responsible for 47% of all cases of under-5 mortality [2]. Neonatal infections are the third most common cause of death during the neonatal period, with 15% of neonatal deaths related to neonatal sepsis [2]. Sepsis-related mortality varies considerably in different regions of the world with the highest incidence and case fatality rate being recorded in areas with the lowest socio-demographic index [3]. Antimicrobial resistance is estimated to be responsible for 126,000 deaths per year worldwide [1]. The global prevalence of antibiotic resistance (ABR) is increasing, and this poses a problem for sepsis treatment in neonatal units [4–9], because the resistance rate of third-generation cephalosporins varies from 26 to 84% for gram-negative bacteria [4]. The World Health Organization (WHO) recommends the use of ampicillin or penicillin + gentamicin [10, 11] as first-line treatment for neonatal sepsis, and third-generation cephalosporins as second-line treatment; when a staphylococcal infection is suspected, flucloxacillin is added to the second-line treatment [11]. Assessing the prevalence of local pathogens and examining their ABR patterns are essential for developing and optimizing local protocols for neonatal sepsis management. In sub-Saharan Africa, limited evidence is available regarding neonatal microbiology and ABR [3, 9], despite the high burden of neonatal infections in the region. Pathogens that are most frequently isolated from blood cultures in African neonates with sepsis include *Staphylococcus aureus*, *Klebsiella* spp. and *Escherichia coli* [9]. Resistance to the WHO-recommended beta-lactams and aminoglycosides in African region are reported in 68 and 27% of cases, respectively [9]. As of 2019, the Central-African Republic (CAR) had the second highest neonatal mortality rate worldwide (41 deaths/1000 live births) [2]. Many reproductive health needs of the population are not met as there is only 0.3 nurses/midwives per 1000 population and only 40% of births are attended by skilled healthcare personnel [12]. Data on microbiology and ABR in the CAR are sporadic, with most data focusing on the ABR of the Enterobacteriaceae, and are rarely specific to the paediatric population [13–17]. To our knowledge, no studies have been published on neonatal infections and their ABR in CAR. Moreover, according to available evidence, there seems to be a high variability in pathogen frequency and resistance patterns between and within different regions in sub-Saharan Africa [9]. This suggests that CAR-specific pathogens and ABR patterns may be very different from those that have been described for the same geographical region and that it would be misleading to make assumptions on pathogen prevalence and their ABR patterns based on available evidence without empirical CAR-specific data. Thus, this study aimed to describe the aetiology, ABR patterns of isolated bacteria, and factors associated with positive blood culture and fatal outcomes in neonates admitted in Castor’s neonatal unit with suspected antibiotic-resistant neonatal infection.

## Methods

### Study design and setting

This hospital-based retrospective cohort study was conducted at the neonatal unit of Castor’s Maternity Bangui, CAR. It included all newborn with “suspected antibiotic-resistant neonatal infection”, as defined in operational definitions, who were admitted in the neonatal unit of Castor’s Maternity from December 2018 to March 2020. Neonates were included regardless of the timing of their infection (early or late), birth weight, and gestational age. Preterm neonates admitted since the first week of life were also included, even if they were older than 28 days at the moment of suspected antibiotic-resistant neonatal infection. In Box 1, we list the operational definitions that were used in this study.

**Box 1:** Operational definitions of neonatal infection-related terms

Neonatal sepsis	Systemic condition of infectious origin, associated with haemodynamic changes and other clinical manifestations, resulting in substantial morbidity and mortality in neonates (children aged less than 28 days) [20].
Diagnostic criteria of neonatal sepsis	According to Médecins Sans Frontières (MSF) neonatal guidelines used in Castor’s neonatal unit, sepsis is diagnosed if one or more of the following signs are present: Fever (> 38°), hypothermia (< 35,5°), poor sucking, haemodynamic instability, apnoea, respiratory distress, cyanosis, grunting, abdominal distension, coma, lethargy, irritability, bulging fontanelles, convulsions, recurrent hypoglycaemia, and purpura-like cutaneous rash.
Clinical neonatal sepsis	Clinical manifestations consistent with infection in neonates with negative cultures of blood and other sterile fluids [33].
Serious bacterial infection	Classically includes bacteraemia, meningitis, urinary tract infection and occasionally pneumonia, enteritis, skin and soft tissue infections [34]. In this paper, “serious bacterial infection” includes bacteraemia, meningitis, pneumonia, and omphalitis.
Neonatal infection	Clinical neonatal sepsis and/or serious bacterial infection.
Suspected antibiotic-resistant neonatal infection	“Neonatal infection” in patients who received antibiotics (first- or second-line), (whichever diagnosis was recorded at discharge) and tested with blood culture because of treatment failure (poor clinical response after 48 h of antibiotic treatment).
Onset of neonatal infection	Early: blood culture taken before 5 days of life (72 h of onset of symptoms + 48 h of treatment failure) Late: blood culture taken after 5 days of life (72 h of onset of symptoms + 48 h of treatment failure)
Birth weight categories	Normal birth weight: > 2500 g Low birth weight (LBW): 1500–2499 g [35–37] Very low birth weight (VLBW): 1000–1499 g [35, 37] Extremely low birth weight (ELBW): < 1000 g [35, 37].
Preterm birth categories	Preterm birth: <37th week of gestation [35, 38, 39]. Late preterm birth: 32nd-37th week of gestation [38, 39]. Very preterm birth: 28th-32nd week of gestation [38, 39]. Extremely preterm birth: < 28th week of gestation [35, 38, 39].
Diagnosis	As recorded by the clinician who filled the chart at discharge.
Preterm-related condition	All “diagnoses” that are primarily related to preterm birth, including necrotizing enterocolitis and apnoea of prematurity syndrome.
Fatal outcome/death	In-hospital death.
ABR prevalence	The number of resistant bacteria isolated divided by the number of bacteria isolated and tested with the drug susceptibility test for a specific antibiotic.
Presence of coagulase-negative *Staphylococci* (CoNS) in blood culture	Considered a contaminant or aetiological agent of neonatal infection according to the information recorded as the final diagnosis in the clinical file.
African regions (central, eastern, western and southern)	As per the African Union definition and used by Okomo et al. [9].

In Bangui, the capital city of CAR, there are only four comprehensive emergency obstetric and new-born care facilities, and Castor’s Maternity offers free healthcare 24 h a day every day. Castor’s Maternity is the most visited maternity clinic in Bangui with an average of 850 deliveries and 170 neonatal admissions per month in 2019. Moreover, in 2019, Castor’s Maternity covered 51% of the expected number of caesarean sections and 72% of the expected number of direct obstetric complications in Bangui. Castor’s neonatal unit is the second-largest unit in Bangui, after the neonatal unit of the paediatric complex of the General Hospital. The unit provides standard neonatal care with a possibility of administering oxygen therapy, intravenous fluids, intravenous drugs, as well as feeding support for preterm neonates with nasogastric tubes. However, as frequently happens in resource limited settings, invasive and non-invasive ventilation, incubators, and central lines are not available in the facility. Neonatal sepsis is treated in Castor’s neonatal unit with ampicillin + gentamicin and cefotaxime as first- and second-line treatments respectively; moreover, since 2017, meropenem (with or without amikacine) has been used as third-line treatment. The Médecins Sans Frontières (MSF) operational centre in Brussels has been supporting Castor’s Maternity since June 2014. In April 2017, an outbreak of extended spectrum beta-lactamase producer *Klebsiella pneumoniae* was detected in Castor’s Maternity, which included 20 confirmed cases and 5 deaths. In response to the outbreak, a comprehensive action plan that included routine blood culture implementation for neonates with neonatal infection and treatment failure was set up in the neonatal unit. MSF continued performing blood culture untill 2020 and thereafter. According to local clinical guidelines, blood culture was not routinely performed for neonates with a clinical diagnosis of neonatal infection but only for those with suspected antibiotic-resistant neonatal infection.

### Data collection

We used a general dataset of all admitted neonates and a dataset of suspected antibiotic-resistant neonatal infections to collect the following variables: date of birth, birth weight, gestational age at birth (assessed with the date of last menstrual period), sex, type of delivery, presence of risk factors for neonatal sepsis (meconial liquid, maternal fever, prolonged membranes rupture, and maternal antibiotic treatment), data of clinical presentation of infection in neonates (persistent fever, jaundice, abdominal distension, skin rash, and resuscitation at birth), final diagnosis, date and result of the blood culture (positive or negative, type of pathogen and its antibiotics susceptibility), discharge date, and outcome at discharge (dead or alive). Specimens for culture (2 mL of blood) were collected via venous or arterial puncture using standard aseptic techniques, and inoculated directly into bottles produced in Bangui Pasteur Institute containing brain-heart-based infusion broth (40 mL). An MSF car transported the samples daily from the hospital to the Pasteur Institute for analysis. At the Institute, the blood culture bottles were incubated at 37 °C for 48 h followed by a systematic inoculation in bromocresol purple agar and chocolate agar. The absence of growth was declared 24 h after inoculation (72 h after incubation). Bacteria were identified using the API 20E Gallerie and API Web Standalone 1.3.2 software. Antibiotic susceptibility was tested using the disc diffusion method on Mueller-Hinton agar (antibiotic discs manufactured by Bio-Rad, Marnes-la-Coquette, France) and interpreted according to the 2019 recommendations of the CA-SFM (Comité de l’Antibiogramme de la Société Française de Microbiologie).

### Data analysis

Frequencies and proportions were calculated for categorical variables. Chi-square or Fisher’s exact tests were used to determine the association between categorical variables. Continuous variables were described using mean and standard deviation if normally distributed and median and interquartile range if non-normally distributed. T-student and Mann-Whitney U test were performed for continuous variables according to their normal or skewed distribution. Fatal outcome was analysed as a categorical variable with risk estimations and risk ratios (RR) calculation. Poisson’s regression, with a robust method of calculation of standard errors, was used to obtain adjusted relative risk (aRR). The model was fitted by adding all variables that are well-established risk factors for mortality (sex, birthweight, gestational age, onset of sepsis, positive blood culture, diagnosis of asphyxia, resuscitation at birth) [18, 19]. We successively excluded variables with higher *p*-values, one after the other, and retained those with p-values < 0.10. A p-value of < 0.05 was considered statistically significant. Statistical analyses were performed using STATA version 15.1 for MacOS (StataCorp, College Station, Texas 77,845 USA).

### Ethics approval and consent to participate

The study relied entirely on routine clinical and laboratory data. The need for informed consent was waived by the Central African Republic Ethic Review Board (Comité éthique et scientifique de la faculté des sciences de la santé de l’Université de Bangui) (UB/FACCS/IPB/CES/20) and Médecins Sans Frontières (MSF) ethics review board (ERB), since the clinical care, the blood culture and the data collected were done as per the routine clinical activity and there was no direct health-related risk to the participants. Describing isolated pathogens and the proportion of ABR resistance could not harm the study population. All data were de-identified from the routine database used in Castor’s Maternity and shared with the investigators in a password-protected file, which could only be accessed by the principal investigator and one other investigator. All experimental protocols were approved by the CAR ERB “Comité éthique et scientifique de la faculté des sciences de la santé de l’Université de Bangui” (UB/FACCS/IPB/CES/20). At MSF, this research fulfilled the exemption criteria set by the MSF ERB for a posteriori analyses of routinely collected clinical data, and thus did not require MSF ERB review. It was conducted with permission from the Medical Director of the Operational Centre Brussels-MSF. The followed procedures were in accordance with the ethical standards of the Helsinki Declaration (2008 amendment) of the World Medical Association.

## Results

### Characteristics of neonates with suspected antibiotic-resistant neonatal infection

During the study period, 2365 patients were admitted to Castor’s neonatal unit. One-hundred-twenty-six blood cultures were performed for 124 patients with suspected antibiotic-resistant neonatal infection. For two patients, blood cultures were performed as follow up after a first positive result to prove the efficacy of treatment. Blood cultures were positive for 33 (26.6%) of 124 patients tested. Eighty-one patients (65.3%) had early onset neonatal infection (EONI) and 41 (33.1%) had late onset neonatal infection (LONI). Table 1 summarises the general demographics and clinical characteristics of the study population according to the onset of neonatal infection. The percentage of positive blood cultures was higher in neonates with LONI than in those with EONI (46.3% versus 17.9%, *p*-value = 0.002; odds ratio 3.9, 95% confidence interval [CI] = 1.6–9.6). Neonates with EONI were more likely to be resuscitated at birth (57.1% versus 35.0%, *p*-value 0.003), have meconium-stained amniotic fluid at birth (46.2% versus 25.0%, p-value = 0.03), and have a diagnosis of asphyxia (40.0% versus 14.6%, *p*-value 0.007). The occurrence of EONI was more likely associated with maternal fever/chorioamnionitis (12.2% versus 0%, *p*-value = 0.03) and a final “diagnosis” (as per the operational definition) of neonatal infection (93.7% versus 75.6%, *p*-value 0.007). In contrast, neonates with LONI were more likely to have a diagnosis of preterm-related condition (51.2% versus 13.7%, *p*-value< 0.001) and abdominal distension (21.0% versus 5.0%, *p*-value = 0.02), which can be a clinical sign of necrotizing enterocolitis. Moreover, neonates with LONI were more likely to present with low birth weight (LBW) (65.9% versus 30.0%, p-value < 0.001) and have preterm birth (70.0% versus 36.2%, p-value 0.002) than those with EONI.

**Table 1:** General characteristics of neonates with suspected antibiotic-resistant neonatal infection admitted to Castor’s neonatal unit (Bangui, CAR) from December 2018 to March 2020

Neonatal characteristics	Early-onset (*n* = 81)	Late-onset (*n* = 41)	Total*	Fisher’s exact test *p*-value
**Sex (*n*** **= 123)**				
Male	41 (51.2%)	18 (43.9%)	60 (48.8%)	0.56
Female	39 (48.7%)	23 (56.1%)	63 (51.2%)	
**Gestational age (*n*** **= 122)**				
Extremely preterm (< 28 weeks)	2 (2.5%)	5 (12.5%)	7 (5.7%)	0.002
Very preterm (28-32 weeks)	11 (13.7%)	12 (30.0%)	25 (26.2%)	
Late preterm (32–37 weeks)	16 (20.0%)	11 (27.5%)	27 (48.4%)	
Normal term (> 37 weeks)	51 (63.7%)	12 (30.0%)	63 (51.6%)	
**Birth weight (n = 123)**				
Extremely low birth weight (< 1000 g)	1 (1.2%)	2 (4.9%)	3 (2.4%)	< 0.001
Very low birth weight (1000–1500 g)	5 (6.2%)	14 (34.1%)	20 (16.3%)	
Low birth weight (1500–2500 g)	18 (22.5%)	11 (26.8%)	30 (24.4%)	
Normal birth weight (> 2500 g)	56 (70.0%)	14 (34.1%)	70 (56.9%)	
Diagnosis** (n = 123)				
Preterm-related condition	11 (13.7%)	21 (51.2%)	32 (26.0%)	< 0.001
Perinatal asphyxia	32 (40.0%)	6 (14.6%)	39 (31.7%)	0.007
Sepsis/serious bacterial infection	75 (93.7%)	31 (75.6%)	106 (86.2%)	0.007
**Outcome (*n*** **= 124)**				
Death	17 (21.0%)	9 (21.9%)	27 (21.8%)	0.004
Cured	61 (75.3%)	24 (58.5%)	86 (69.3%)	
Referred	0 (0%)	6 (14.6%)	6 (4.8%)	
Evaded/unknown	3 (3.7%)	2 (4.9%)	5 (4.0%)	
**Blood culture result (*n*** **= 120)**				
Positive	14 (17.9%)	19 (46.3%)	33 (27.5%)	0.002
Negative	64 (82.0%)	22 (53.7%)	87 (72.5%)	
**Clinical manifestations**				
Persistent fever (*n* = 119)	66 (82.5%)	24 (61.5%)	90 (75.6%)	0.02
Jaundice (n = 119)	16 (20.0%)	13 (33.3%)	29 (24.4%)	0.12
Abdominal distension (*n* = 118)	4 (5.0%)	8 (21.0%)	12 (10.2%)	0.02
Rash (n = 118)	4 (5.0%)	4 (10.5%)	8 (6.8%)	0.27
Resuscitation at birth (*n* = 117)	44 (57.1%)	14 (35.0%)	60 (50.4%)	0.03
**Delivery characteristics**	**Early-onset (n = 81)**	**Late-onset (n = 41)**	**Total**	**Fisher’s exact test p-value**
**Delivery type (n = 123)**				
Vaginal	55 (68.7%)	31 (75.6%)	88 (71.5%)	0.17
Vacuum-assisted	3 (3.7%)	4 (9.8%)	7 (5.7%)	
Caesarean	22 (27.5%)	6 (14.6%)	28 (22.8%)	
**Place of delivery (*n*** **= 110)**				
Other than Castor’s health facility	0 (0%)	2 (5.4%)	2 (1.8%)	0.11
**Infectious risk factors**				
Maternal fever/chorioamnionitis (*n* = 113)	9 (12.2%)	0 (0%)	9 (8.0%)	0.03
Prolonged membrane rupture (> 18 h) (n = 123)	27 (33.7%)	10 (24.4%)	38 (30.9%)	0.40
Meconial amniotic liquid (n = 122)	37 (46.2%)	10 (25.0%)	48 (39.3%)	0.03
Maternal antibiotic treatment (*n* = 107)	22 (31.2%)	4 (11.1%)	27 (25.2%)	0.03

*We found 2 patients with unknown infection onset (missing blood culture date), which explains the mismatch between the total number of infections and the sum of the number of early- and late-onset neonatal infections

**The diagnostic categories are not mutually exclusive

### Microbiology of isolated pathogens

Table 2 summarises the pathogens isolated from the blood cultures of neonates with suspected antibiotic-resistant neonatal infection. Among 33 patients with positive blood culture, the vast majority of isolated bacteria were gram-negative bacteria (87.9%). The most frequently isolated gram-negative bacteria were *Klebsiella pneumoniae* (44.8%), *Escherichia coli* (24.1%) and *Klebsiella oxytoca* (20.7%). In neonates with EONI, *Klebsiella pneumoniae* and *Escherichia coli* were the most frequent gram-negative pathogens (41.7% each), and in those with LONI, *Klebsiella pneumoniae* was the most frequent gram-negative pathogen (47%), followed by *Klebsiella oxytoca* (35.3%) and *Escherichia coli* (11.8%). We found 4 gram-positive bacteria: 2 *Staphylococcus aureus* and 2 Coagulase negative Staphylococci (CoNS). The 2 patients with CoNS isolated in blood culture received first and seond-line treatment. They both recovered, one was considered in the final diagnosis infected with CoNS, for the other we did not know if the CoNS was considered pathogen or contaminant.

**Table 2:** Isolated pathogens with respect to infection onset in neonates with suspected antibiotic-resistant neonatal infection admitted to Castor’s neonatal unit (Bangui, CAR) from December 2018 to March 2020

	Early-onset		Late-onset		All positives	
	***n*** = 14	%	***n*** = 19	%	***n*** = 33	%
**Gram-negative**	**12**	**85.7**	**17**	**89.5**	**29**	**87.9**
*Klebsiella pneumoniae*	5	41.7	8	47.0	13	44.8
*Escherichia coli*	5	41.7	2	11.8	7	24.1
*Klebsiella oxytoca*	0	0	6	35.3	6	20.7
*Enterobacter cloacae*	1	8.3	1	5.9	2	6.9
*Salmonella spp*	1	8.3	0	0	1	3.4
*Total (gram-negative)*	12	100	17	100	29	100
**Gram-positive**	**2**	**14.3**	**2**	**10.5**	**4**	**12.1**
*Staphylococcus aureus*	1	50	1	50	2	50
Coagulase-negative staphylococci	1	50	1	50	2	50
*Total (gram-positive)*	2	100	2	100	4	100

Table 3 summarises the proportions of antibiotics resistance observed in the most commonly isolated pathogens and in gram-negative bacteria all together. Additional files 2 and 3 split Table 3 according to onset of neonatal infection (early for Additional file 2 and late for Additional file 3). None of the tested bacteria were sensitive to ampicillin. Resistance to gentamicin was observed in 100% of isolated *Klebsiella pneumoniae*, 80% of tested *Klebsiella oxytoca* and 85.7% of isolated *Escherichia coli*. Both isolated *Staphylococcus aureus* and half of the two strains of isolated *Enterobacter cloacae* were resistant to gentamicin. Resistance to third generation cephalosporins was observed in 100% of tested *Klebsiella pneumoniae*, 83.3% of isolated *Klebsiella oxytoca*, 50% of tested *Escherichia coli* and 50% of isolated *Enterobacter cloacae*. Only 14.3% of all gram-negative tested bacteria were sensitive to first-line treatment (ampicillin + gentamicin) and 22.2% were sensitive to second-line treatment (third generation cephalosporins). Moreover, resistance to amikacin was observed in 33.3% of isolated *Klebsiella oxytoca* and in none of the other tested bacteria. Reduced susceptibility to ciprofloxacin was observed in 88.9% of tested *Klebsiella pneumoniae*, 83.3% of isolated *Klebsiella oxytoca*, 80% of tested *Escherichia coli* and 100% of isolated *Enterobacter cloacae.* Only 13.6% of the gram-negative tested bacteria were sensitive to ciprofloxacin. None of the tested bacteria was resistant to carbapenems. Among the two isolated *Staphylococcus aureus* strains, one was resistant to cefoxitin (both tested) and one was resistant to ciprofloxacin (only one tested). Additional file 1 (in additional files) shows the resistance profile of each pathogen isolated in our study population.

**Table 3:** Isolated gram-negative pathogens and susceptibility to antibiotics in neonates with suspected antibiotic-resistant neonatal infection admitted to Castor’s neonatal unit (Bangui, CAR) from December 2018 to March 2020

Antibiotic	*K.pneumoniae* (*n* = 13)		*K.oxytoca* (*n* = 6)		*E.coli* (*n* = 7)		*All gram-negative bacteria* (*n* = 29)	
	R/(R + S)	R%	R/(R + S)	R%	R/(R + S)	R%	R/(R + S)	R%
**Beta-lactam**								
AMP	13/13	100%	6/6	100%	6/6	100%	27/27	100%
FOX	1/13	7.7%	2/6	33.3%	1/7	14.3%	5/29	17.2%
CTX	12/12	100%	5/6	83.3%	3/6	50%	21/27	77.8%
FEP	12/12	100%	5/6	83.3%	4/7	57.1%	21/28	75%
IPM	0/12	0%	0/6	0%	0/7	0%	0/28	0%
**Non-beta-lactam**								
GEN	13/13	100%	4/5	80%	6/7	85.7%	24/28	85.7%
AMK	0/13	0%	2/6	33.3%	0/7	0%	2/29	6.9%
CAF	3/12	25%	5/6	83.3%	2/7	28.6%	13/28	46.4%
	**(R + I)/(R + I + S)**	**(R + I)%**	**(R + I)/(R + I + S)**	**(R + I)%**	**(R + I)/(R + I + S)**	**(R + I)%**	**(R + I)/(R + I + S)**	**(R + I)%**
CIP	8/9	88.9%	5/5	100%	4/5	80%	19/22	86.4%

AMK = Amikacin; AMP = Ampicillin; CAF=Chloramphenicol CIP=Ciprofloxacin; CTX = Cefotaxime; FEP=Cefepime; FOX = Cefoxitin; GEN = Gentamicin; I=Intermediate; IPM = Imipenem; R = Resistant; S=Sensitive

### Factors associated with fatal outcome

Table 4 shows the RR for death after being adjusted for the blood culture result, birth weight, sex, and diagnosis of asphyxia. Neonates with positive blood culture had a higher risk of death (38.7%) than those with negative blood culture (19%) (aRR = 2.32; 95%CI = 1.17–4.60). Male neonates had a statistically significantly higher risk of death than female neonates (aRR = 2.07; 95%CI = 1.01–4.26). The risk of death was higher in neonates with a final diagnosis of asphyxia, than those without (aRR = 2.42; 95%CI = 1.07–5.47). Neonates with birth weight < 2500 g had a higher risk of death (aRR = 2.21; 95% CI = 1.0–4.91, aRR = 2.74; 95% CI = 1.3–5.79, and aRR = 2.31; 95% CI = 1.0–4.91 for LBW, VLBW, and ELBW, respectively).

**Table 4:** Poisson regression analysis of case fatality ratio adjusting for blood culture result, presence of asphyxia, birth weight and sex, in neonates with suspected ABR infection admitted to Castor’s neonatal unit (Bangui, CAR) from December 2018 to March 2020

Death	Adjusted Relative Risk	Standard Error (Robust)	95% Confidence interval	
**Birthweight**				
Extremely low birth weight (< 1000 g)	2.31	2.96	0.19	28.31
Very low birth weight (1000–1499 g)	2.74	1.05	1.30	5.79
Low birth weight (1500–2499 g)	2.21	0.90	1.00	4.91
**Sex**				
Male	2.07	0.76	1.01	4.26
**Diagnosis**				
Asphyxia	2.42	1.01	1.07	5.47
**Blood culture**				
Positive result	2.32	0.81	1.17	4.60
Constant	0.057	0.024	0.024	0.132

Note: Constant estimates baseline risk

## Discussion

In 26.6% of neonates with suspected antibiotic-resistant neonatal infection in Castor’s neonatal unit, an underlying pathogen was identified from blood culture, with gram-negative bacteria (*Klebsiella pneumoniae Klebsiella oxytoca* and *Escherichia coli*) accounting for over 80% of the identified pathogens. All the identified pathogens were resistant to at least one antibiotic. All the tested bacteria were resistant to ampicillin, and more than 80% of the gram-negative tested pathogens were resistant to both antibiotics of the ampicillin-gentamicin combination, which is the first line treatment for neonatal sepsis as per the WHO [10, 11] and MSF guidelines. Nearly 80% of these gram-negative tested pathogens were also resistant to third-generation cephalosporines, which is the second (empirical) choice for neonatal sepsis not responding to first-line treatment. None of the tested bacteria were resistant to carbapenems. Overall, we found that 77.8% of neonates with confirmed gram-negative sepsis could no longer be effectively treated without wide-spectrum antibiotics that are not routinely used in referral hospitals in CAR. Fatal outcome was recorded in 38.7 and 19% of patients with positive and negative blood cultures, respectively. After adjusting for birth weight, sex, blood culture result, and the diagnosis of asphyxia, the risk of death increased in neonates with positive blood culture, male sex, diagnosis of asphyxia, and low birth weight.

In our study population, patients with LONI were more likely to have a positive culture result than those with EONI. This is probably because the LONI group had a higher proportion of preterm neonates and neonates with LBW, in whom the incidence of infection is 3–10 times higher than that in full-term neonates with normal birth weight [20]. The difference in gestational age and birth weight between neonates with EONI and LONI is linked to our selection criteria. We included only hospital-admitted neonates who required medical care in addition to essential new-born care. Full-term neonates with normal birth weight are more likely to receive essential new-born care and get discharged before LONI can develop. This hypothesis is corroborated by the fact that neonates with LONI were more likely to have a diagnosis of preterm-related conditions (and abdominal distension, which is a frequent clinical sign in necrotizing enterocolitis), whereas neonates with EONI were more likely to have had diagnosis of asphyxia, resuscitation at birth, and meconium stained amniotic fluid at birth (common in neonates with asphyxia).

The proportion of neonates with positive blood cultures (26.6%) in our study population (suspected antibiotic-resistant neonatal infection) is similar to that in a previously described sub-Saharan neonatal cohort (25, 95% CI = 21–29%) [9]; however, it is much higher than that reported in a cohort in Delhi, India (6.2, 95% CI = 5.8–6.6%) [21]. This difference might be because we included only neonates with suspected antibiotic-resistant neonatal infection or because of the geographical variation in the incidence of neonatal sepsis [3]. Gram-negative bacteria (mostly *Klebsiella pneumoniae, Klebsiella oxytoca* and *Escherichia coli*) were isolated in 87.9% of positive culture samples, which is higher than that described in the central African region (*Klebsiella spp* 34%, *Escherichia coli* 17% and *Staphylococcus aureus* 26%) [9]. These differences are even more marked when compared with the entire sub-Saharan region (*Klebsiella spp.* 21%, *Escherichia coli* 10% and *Staphylococcus aureus* 25%) [9]. Group B *Streptococcus* (GBS) was not isolated in our retrospective cohort, whereas 6–8% of GBS was reported in the literature in the central African region [9]. We hypothesised that this was because we tested neonates who were being treated with beta-lactam antibiotics and because GBS resistance to beta-lactam agents is still considered infrequent [22]; however, it is estimated that approximately 25% of GBS strains in sub-Saharan Africa are resistant to ampicillin and ceftriaxone [23]. Unfortunately, mothers are not screened for GBS in Castor’s maternity and we do not know the prevalence of GBS colonisation. The high frequency of gram-negative nosocomial bacteria and low frequency of classical aetiological agents of EONI in our study may be linked to either an early horizontal transmission between admitted neonates or a vertical transmission from the maternal genital tract colonised with nosocomial flora due to inadequate infection prevention and control measures. In our study, the resistance of gram-negative bacteria to third-generation cephalosporins and ampicillin was similar to that reported in previous studies conducted in the central African region [9]. On the other hand, gentamicin resistance was considerably higher in gram-negative tested strains in our study as compared to previous studies conducted in the central African region (94.4% versus 86% for *Klebsiella spp.* and 85.7% versus 26% for *Escherichia coli*) [9]. Carbapenems resistance is typically observed to be low in the central African region (11% of *Klebsiella spp.* and 0% of *Escherichia coli*) [9], as is amikacin resistance (19% of *Klebsiella spp.* and 0% of *Escherichia coli*) [9]. We found no carbapenems- or amikacin-resistant strains of *Escherichia coli* and *Klebsiella pneumoniae* in our study population. *Klebsiella oxytoca* showed no carbapenems resistance but was resistant to amikacin in 33.3% of cases. In sub-Saharan Africa, ciprofloxacin resistance varies widely, with ciprofloxacin resistance of *Klebsiella spp*. ranging from 14% in the east African region to 92% in the central African region and that of *Escherichia coli* ranging from 4% in the east African region to 64% in the southern African region [9]. In our population, we report reduced susceptibility to ciprofloxacin (both resistant and intermediate), which makes it difficult to compare our results with those of other authors; however no specific data are reported regarding ciprofloxacin resistance in neonates in the central African region [9].

The case fatality rates of all neonates with suspected antibiotic-resistant neonatal infection (23.9%) and those with blood culture-confirmed sepsis (38.7%) were slightly lower than the case fatality rates reported by other authors (neonatal sepsis = 26% confirmed neonatal sepsis = 48%) [21]. This suggests that the quality of care in our neonatal ward was quite good. Others sub-Saharan cohorts have reported lower [24] or higher [25] case fatality rates; however, they also had concurrent lower [24] or higher [25] proportions of positive blood cultures and ABR prevalence, which may explain the observed differences in mortality. As already described in the literature [18, 19, 21, 26, 27], our findings emphasised how the male sex, a diagnosis of asphyxia, and blood culture-confirmed neonatal sepsis were associated with an increased risk of neonatal death. The presence of gram-negative bacteria in neonatal blood cultures has been previously associated to higher mortality rates in large sub-Saharan neonatal cohorts [24], a trend that is consistent with our results.

### Limitations of the study

This study has some limitation. First, we had a small number of isolated bacteria. However, the currently accepted estimation of the aetiology and prevalence of ABR in central African region reported by Okomo et al. [9] is based on 90 bacterial strain isolated in blood cultures from 4 studies (22 gram-positive and 68 gram-negative, with only 17 *E. coli* and 38 *Klebsiella spp*.); therefore, our study can add to this sparse evidence despite its relatively small number of isolated bacteria. Furthermore, other studies from the central-African region are also similarly small scaled [28–31]. Second, our definition of suspected antibiotic-resistant neonatal infection included the notion of treatment failure defined as poor clinical evolution after 48 h of antibiotic treatment. Thus, we only studied neonates who presented with an evidence of treatment failure, which might have led to an overestimation of the ABR prevalence and case fatality. On the other hand, severely ill patients did not survive long enough (beyond 48 h) to undergo blood culture, which might have led to case fatality rate and ABR prevalence underestimation. Moreover, since we cultured blood from neonates who had already been treated with antibiotics, the prevalence of positive blood cultures could have been underestimated; however, this is not in line with the quite high prevalence of positive blood cultures we found in our cohort. Therefore, the external validity of our results is limited to neonates who had a diagnosis of neonatal infection, presented poor clinical response after 48 h of antibiotic treatment, and were admitted to a neonatal unit in sub-Saharan Africa. Third, we did not have follow up information after discharge, and we studied a neonatal population with a consistent number of preterm deliveries and LBW in a high-neonatal-mortality setting [2]. Consequently, we cannot assume that the cured patients survived at home after being discharged, therefore, our case fatality rates are only valid for the period of hospitalisation. However, this limitation does not affect our results regarding the frequencies of pathogens and ABR prevalence. Fourth, based on the assumption that the neonate’s blood was cultured neither before nor after 48 h of treatment failure, we selected the cut-off value of 5 days of life at the moment of blood sample collection to separate EONI from LONI; therefore, the cut-off value for the onset of signs and symptoms was 72 h, which has been considered previously by a majority of authors [20]. However, we cannot defend this time selection because some neonates with EONI who developed symptoms after 72 h of life could have been tested with blood culture before the 5th day of life. Also, some neonates who developed symptoms within the first 72 h of life could have been tested after the 5th day of life. Hence, it is impossible to make an onset-specific comparison of our findings with those of other studies. Fifth, we could not report gestational age-specific case fatality rates estimates because gestational age estimation in CAR setting is not always reliable. We preferred to not calculate the RR adjusted for gestational age categories, but we rather used the birth weight as a proxy of gestational term for our analysis. This approach has previously shown limitations [32]; therefore, we consider this to be a disadvantage in our study.

## Conclusion

A major finding of this study was the isolation of gram-negative bacteria (mostly *Klebsiella pneumoniae Klebsiella oxytoca* and *Escherichia coli*) in almost 9 of 10 positive samples (with similar percentages in EONI and LONI), a proportion that is higher than what has been described elsewhere in sub-Saharan Africa. We also detected a comparatively higher profile of gentamicin resistance in our study population, and most of the isolated pathogens showed resistance to first- and second-line treatments. This study highlights that carbapenem could be considered as second line treatment for neonatal infection in critically ill patients. Blood cultures should be done prior to commencing new antibiotics. However further larger, multicentered studies are warranted in low middle-income countries. For settings that are similar to those of Castor’s Maternity (with similar bacterial profiles and resistance patterns), we recommend carbapenem use as second-line treatments for neonatal sepsis, at least for specific groups of patients. Future studies are warranted to identify such groups. We further recommend that ABR prevalence in neonates with first-line treatment failure be studied routinely to further inform the medical community about the use of carbapenems as a possible second-line option. Carbapenems should not be used as empirical routine second- or third-line treatment options in facilities where blood culture is not routinely performed in neonates with failure of first and/or second line antibiotic treatment.

## Supplementary Information

**Additional file 1.**

**Additional file 2.**

**Additional file 3.**

## Data Availability

The dataset generated or analysed can be made available to interested researchers by the authors of this article under previous MSF-OCB permission.

## Abbreviations

AMK: Amikacin
AMP: Ampicillin
ABR: Antibiotic resistance
aRR: Adjusted Relative risk
CAF: Chloramphenicol
CAR: Central-African Republic
CIP: Ciprofloxacin
CoNS: Coagulase-negative staphylococci
CRO: Ceftriaxone
CTX: Cefotaxime
ELBW: Extremely low birth weight
EONI: Early-onset neonatal infection
ERB: Ethics Review Board
FEP: Cefepime
FOX: Cefoxitin
GBS: Group B Streptococcus (*Streptococcus agalactiae*)
GEN: Gentamicin
CI: Confidence interval
IPM: Imipenem
LBW: Low birth weight
LONI: Late-onset neonatal infection
MSF: Médecins Sans Frontières
VLBW: Very low birth weight
WHO: World Health Organization
