# Supplementary material for: When first line treatment of neonatal infection is not enough: blood culture and resistance patterns in neonates requiring second line antibiotic therapy in Bangui, Central African Republic
Source: BMC Pediatr. 2021 Dec 13;21:570. doi: 10.1186/s12887-021-02911-w (PMC8667452; doi:10.1186/s12887-021-02911-w)
Supplement: Supplementary file 1 — Additional file 1. [file 12887_2021_2911_MOESM1_ESM.docx]

Additional file 1. Isolated pathogens and susceptibility to antibiotics in neonates with suspected antibiotic-resistant neonatal infection admitted to Castor’s neonatal unit (Bangui, CAR) from December 2018 to March 2020.

| Pathogen | AMP | FOX | FEP | CTX | IMP | GEN | AMK | CAF | CIP |
| --- | --- | --- | --- | --- | --- | --- | --- | --- | --- |
| *K. pneumoniae* 1 | R | S | R | R | S | R | S | S | NA |
| *K. pneumoniae* 2 | R | S | R | R | S | R | S | NA | NA |
| *K. pneumoniae* 3 | R | S | R | R | S | R | S | R | R |
| *K. pneumoniae* 4 | R | S | R | NA | NA | R | S | S | NA |
| *K. pneumoniae* 5 | R | R | R | R | S | R | S | S | R |
| *K. pneumoniae* 6 | R | S | R | R | S | R | S | S | R |
| *K. pneumoniae* 7 | R | S | I | R | S | R | S | R | I |
| *K. pneumoniae* 8 | R | S | R | R | S | R | S | S | R |
| *K. pneumoniae* 9 | R | S | R | R | S | R | S | S | NA |
| *K. pneumoniae* 10 | R | S | R | R | S | R | S | S | R |
| *K. pneumoniae* 11 | R | S | R | R | S | R | S | S | R |
| *K. pneumoniae* 12 | R | S | R | R | S | R | S | R | S |
| *K. pneumoniae* 13 | R | S | R | R | S | R | S | S | R |
| *K. oxytoca* 1 | R | R | R | R | S | R | R | R | R |
| *K. oxytoca* 2 | R | S | R | R | S | R | S | R | NA |
| *K. oxytoca* 3 | R | S | R | R | S | R | S | R | R |
| *K. oxytoca* 4 | R | S | R | R | S | NA | R | R | I |
| *K. oxytoca* 5 | R | R | R | R | S | R | S | S | R |
| *K. oxytoca* 6 | R | S | S | S | S | S | S | R | R |
| *E. coli* 1 | R | S | R | R | S | R | S | S | R |
| *E. coli* 2 | R | S | R | R | S | R | S | S | R |
| *E. coli* 3 | R | S | S | S | S | R | S | S | S |
| *E. coli* 4 | NA | S | R | NA | S | R | S | S | R |
| *E. coli* 5 | R | S | S | S | S | S | S | R | NA |
| *E. coli* 6 | R | R | S | S | S | R | S | R | R |
| *E. coli* 7 | R | S | R | R | S | R | S | S | NA |
| *E. cloacae* 1 | R | R | R | R | S | R | S | R | R |
| *E. cloacae* 2 | NA | S | S | S | S | S | S | R | R |
| *Salmonella sp* | R | S | S | S | S | S | S | R | S |
| *S. aureus* 1 | NA | S | NA | NA | NA | R | NA | NA | NA |
| *S. aureus* 2 | NA | R | NA | NA | NA | R | NA | NA | R |
| CoNS 1 | NA | NA | NA | NA | NA | NA | NA | NA | NA |
| CoNS 2 | NA | NA | NA | NA | NA | NA | NA | NA | R |
|  | | | | | | | | | |
| AMK=Amikacin; AMP=Ampicillin; CAF=Chloramphenicol; CIP=Ciprofloxacin; CoNS=Coagulase negative *Staphylococcus*; CTX=Cefotaxime; FEP=cefepime FOX=Cefoxitin; GEN=Gentamicin; I=Intermediate; IPM=Imipenem; NA=Not tested; R=Resistant; S=Sensitive | | | | | | | | | |
