# Supplementary material for: When first line treatment of neonatal infection is not enough: blood culture and resistance patterns in neonates requiring second line antibiotic therapy in Bangui, Central African Republic
Source: BMC Pediatr. 2021 Dec 13;21:570. doi: 10.1186/s12887-021-02911-w (PMC8667452; doi:10.1186/s12887-021-02911-w)
Supplement: Supplementary file 2 — Additional file 2. [file 12887_2021_2911_MOESM2_ESM.docx]

Additional file 2. *Isolated gram-negative pathogens and susceptibility to antibiotics in neonates with suspected antibiotic-resistant early onset neonatal infection admitted to Castor’s neonatal unit (Bangui, CAR) from December 2018 to March 2020.*

| **Antibiotic** | *K.pneumoniae* (n=5) | | *E.coli* (n=5) | | *All gram-negative bacteria* (n=12) | |
| --- | --- | --- | --- | --- | --- | --- |
|  | R/(R+S) | R% | R/(R+S) | R% | R/(R+S) | R% |
| **Beta-lactam** | |  |  |  |  |  |
| AMP | 5/5 | 100% | 4/4 | 100% | 11/11 | 100% |
| FOX | 1/5 | 20% | 0/5 | 0% | 2/12 | 16.7% |
| CTX | 4/4 | 100% | 2/4 | 50% | 7/10 | 70% |
| FEP | 5/5 | 100% | 3/5 | 60% | 9/12 | 75% |
| IPM | 0/4 | 0% | 0/5 | 0% | 0/11 | 0% |
| **Non-beta-lactam** | |  |  |  |  |  |
| GEN | 5/5 | 100% | 4/5 | 80% | 10/12 | 83.3% |
| AMK | 0/5 | 0% | 0/5 | 0% | 0/12 | 0% |
| CAF | 1/5 | 20% | 1/5 | 20% | 4/12 | 33.3% |
|  | R+I/(R+I+S) | R+I% | R+I/(R+I+S) | R+I% | R+I/(R+I+S) | R+I% |
| CIP | 3/3 | 100% | 3/4 | 75% | 7/9 | 77.8% |
| AMK=Amikacin; AMP=Ampicillin; CAF=Chloramphenicol CIP=Ciprofloxacin; CTX=Cefotaxime; FEP=Cefepime; FOX=Cefoxitin; GEN=Gentamicin; I=Intermediate; IPM=Imipenem; R=Resistant; S=Sensitive | | | | | | |
