# Supplementary material for: When first line treatment of neonatal infection is not enough: blood culture and resistance patterns in neonates requiring second line antibiotic therapy in Bangui, Central African Republic
Source: BMC Pediatr. 2021 Dec 13;21:570. doi: 10.1186/s12887-021-02911-w (PMC8667452; doi:10.1186/s12887-021-02911-w)
Supplement: Supplementary file 3 — Additional file 3. [file 12887_2021_2911_MOESM3_ESM.docx]

Additional file 3. *Isolated gram-negative pathogens and susceptibility to antibiotics in neonates with suspected antibiotic-resistant late onset neonatal infection admitted to Castor’s neonatal unit (Bangui, CAR) from December 2018 to March 2020.*

| **Antibiotic** | *K.pneumoniae* (n=8) | | *K.oxytoca* (n=6) | | *E.coli* (n=2) | | *All gram-negative bacteria* (n=17) | |
| --- | --- | --- | --- | --- | --- | --- | --- | --- |
|  | R/(R+S) | R% | R/(R+S) | R% | R/(R+S) | R% | R/(R+S) | R% |
| **Beta-lactam** | |  |  |  |  |  |  |  |
| AMP | 8/8 | 100% | 6/6 | 100% | 2/2 | 100% | 16/16 | 100% |
| FOX | 0/8 | 0% | 2/6 | 33.3% | 1/2 | 50% | 3/17 | 17.6% |
| CTX | 8/8 | 100% | 5/6 | 83.3% | 1/2 | 50% | 14/17 | 82.4% |
| FEP | 7/7 | 100% | 5/6 | 83.3% | 1/2 | 50% | 13/16 | 82.4% |
| IPM | 0/8 | 0% | 0/6 | 0% | 0/2 | 0% | 0/17 | 0% |
| **Non-beta-lactam** | |  |  |  |  |  |  |  |
| GEN | 8/8 | 100% | 4/5 | 80% | 2/2 | 100% | 14/16 | 87.5% |
| AMK | 0/8 | 0% | 2/6 | 33.3% | 0/2 | 0% | 2/17 | 11.8% |
| CAF | 2/7 | 28.6% | 5/6 | 83.3% | 1/2 | 50% | 9/16 | 46.4% |
|  | R+I/(R+I+S) | R+I% | R+I/(R+I+S) | R+I% | R+I/(R+I+S) | R+I% | R+I/(R+I+S) | R+I% |
| CIP | 5/6 | 83.3% | 5/5 | 100% | 1/1 | 100% | 12/13 | 92,3% |
| AMK=Amikacin; AMP=Ampicillin; CAF=Chloramphenicol CIP=Ciprofloxacin; CTX=Cefotaxime; FEP=Cefepime; FOX=Cefoxitin; GEN=Gentamicin; I=Intermediate; IPM=Imipenem; R=Resistant; S=Sensitive | | | | | | | | |
